# Taking Medicines

**Published:** 1884-10

**Authors:** 


### TAKING MEDICINES.

MISCHIEF is often done by the indiscriminate use of medicines. The idea is well expressed by the inscription on and old tomb-stone :

> "*I was well ; I wished to be better ;*
> *I took physic, and here I am !*"

The intelligent physician does not profess to cure disease through the direct agency of the remedies he prescribes ; these are given to remove obstructions that interfere with the recuperative efforts of nature. If there are no obstructions to remove, the effect of drugs is to interfere with the natural and healthful movements of the machinery of life. Health is maintained by "good living,"—a term that comprehends a great deal. It consists in having good food, properly cooked, at every meal ; clothing appropriate to the changing seasons; and moderation in all things. Such a person might require no medicine during a long life.

I must admit, however, that such an instance would be exceptional, even to one making the effort to live in that way. We can not always procure well-cooked foods, nor can we always predict sudden changes of the weather in time to protect ourselves against them. But we can aid nature in throwing off disease, by abstinence and such other prudential means as would occur to any thoughtful person, instead of eating heartily and trusting in drugs to overcome our ailments.

Who ever saw an habitual medicine-taker who enjoyed reasonably good health ? All medicines debilitate, and that drawback must be duly considered before taking them. Think of the quantities of pills that are used. Most of these are taken to relieve constipation. Unfortunately the relief is only temporary, and the doses must be repeated often, thus weakening the stomach and incapacitating it for its natural work. If medicine is used for the relief of constipation it is better to employ it in *suppositories*, but a better plan than either is to cure the trouble by means of a proper diet and regular and active exercise or work in the open air. Thousands of drunkards, with their legacies of sorrow and crime and broken hearts, are made through dram drinking, commenced at first for the relief of dyspepsia or colic, and continued through excuses and subterfuges that a depraved appetite strives to make plausible.

The little household remedies have their uses, and they have also their abuses. There are occasions when such remedies as camphor, brandy, paregoric, laudanum, ginger, and pills and powders, may be of great service. The important point is to know when to use them ; that would be perhaps once where they are ordinarily employed ten times. The best of all remedies—and every person should have a little constantly on hand—is common sense. If one experiences inconvenience in eating, nature will bring relief sooner and more effectually if left to herself, than by efforts to aid her with liquors and tinctures that benumb the stomach and retard healthy action. Rest, warmth and abstinence are the proper remedies for all ordinary ailments. Wholesome and nutritious food, the comforts of a good home, vigorous and regular exercise, seasonable clothing, fresh air constantly, and eight hours of sound sleep out of every twenty-four, and you may "*throw physic to the dogs.*"

